# Primary non-hepatoblastoma liver tumors in children—Defining the profile of a very rare subset of childhood tumors

**DOI:** 10.1007/s12664-025-01823-2

**Published:** 2025-07-18

**Authors:** Abha Mehta, Badira Cheriyalinkal Parambil, Akshay Baheti, Venkata Ram Mohan Gollamudi, Maya Prasad, Vasundhara Patil, Sajid Qureshi, Mukta Ramadwar, Poonam Panjwani, Kunal Gala, Siddhartha Laskar, Nehal Khanna, Jifmi Jose Manjali, Sneha Shah, Girish Chinnaswammy

**Affiliations:** 1 Division of Pediatric Oncology, Tata Memorial Hospital, Homi Bhabha National Institute, Mumbai, 400 012 India; 2 Department of Radiodiagnosis, Tata Memorial Hospital, Homi Bhabha National Institute, Mumbai, 400 012 India; 3 Department of Pediatric Surgery, Tata Memorial Hospital, Homi Bhabha National Institute, Mumbai, 400 012 India; 4 Department of Pathology, Tata Memorial Hospital, Homi Bhabha National Institute, Mumbai, 400 012 India; 5 Department of Radiation Oncology, Tata Memorial Hospital, Homi Bhabha National Institute, Mumbai, 400 012 India; 6 Department of Nuclear Medicine, Tata Memorial Hospital, Homi Bhabha National Institute, Mumbai, 400 012 India

**Keywords:** Children, Liver tumors, Non-hepatoblastoma, Primary hepatic malignancies

## Abstract

**Background:**

Primary pediatric non-hepatoblastoma (n-HB) liver tumors are rare with limited literature on their clinical-epidemiological profile and outcomes. We audit the above in this study.

**Methods:**

Children diagnosed with n-HB primary liver tumors from January 2012 to December 2023 were analyzed retrospectively. Patients underwent contrast-enhanced computed tomography (CECT) of the abdomen and computed tomography (CT) of the thorax or fluorodeoxyglucosepositron emission tomography/computed tomography (FDG-PET/CT) for staging of malignant tumors. Treatment was administered based on the definitive diagnosis.

**Results:**

Sixty-nine patients formed the study cohort. The most common tumors in various age groups were infantile hepatic hemangioma (IHH)—66.7%, malignant rhabdoid tumor (MRT)—25%; six months to three years: MRT—25.0%, mesenchymal hamartoma and hemangioendothelioma—18.7% each; three to 10 years: hepatocellular carcinoma (HCC)—31.6%, undifferentiated embryonal sarcoma of the liver (UESL)—26.3%; and > = 10 years: HCC—45.4%, UESL—22.7%. Median alpha-fetoprotein (AFP) level in HCC was 131,249 ng/mL. Treatment was delivered to 65.8% patients. Chemotherapy for treated malignant tumors was administered in 81.5%, surgery in 85.2% and radiotherapy in 18.5%, alone or combined. In the different malignant cancer sub-types, the proportion of relapse/deaths in treated patients was HCC—22.2%, UESL—33.3%, rhabdoid—25%, hemangioendotheliomas—25% and sarcomas—25%.

**Conclusions:**

There was a high proportion of malignant rhabdoid tumors and higher serum AFP levels in HCC in our cohort. The overall outcomes of treated malignant tumors were relatively favorable, though limited by the sample size in this rare cohort.

**Graphical Abstract:**

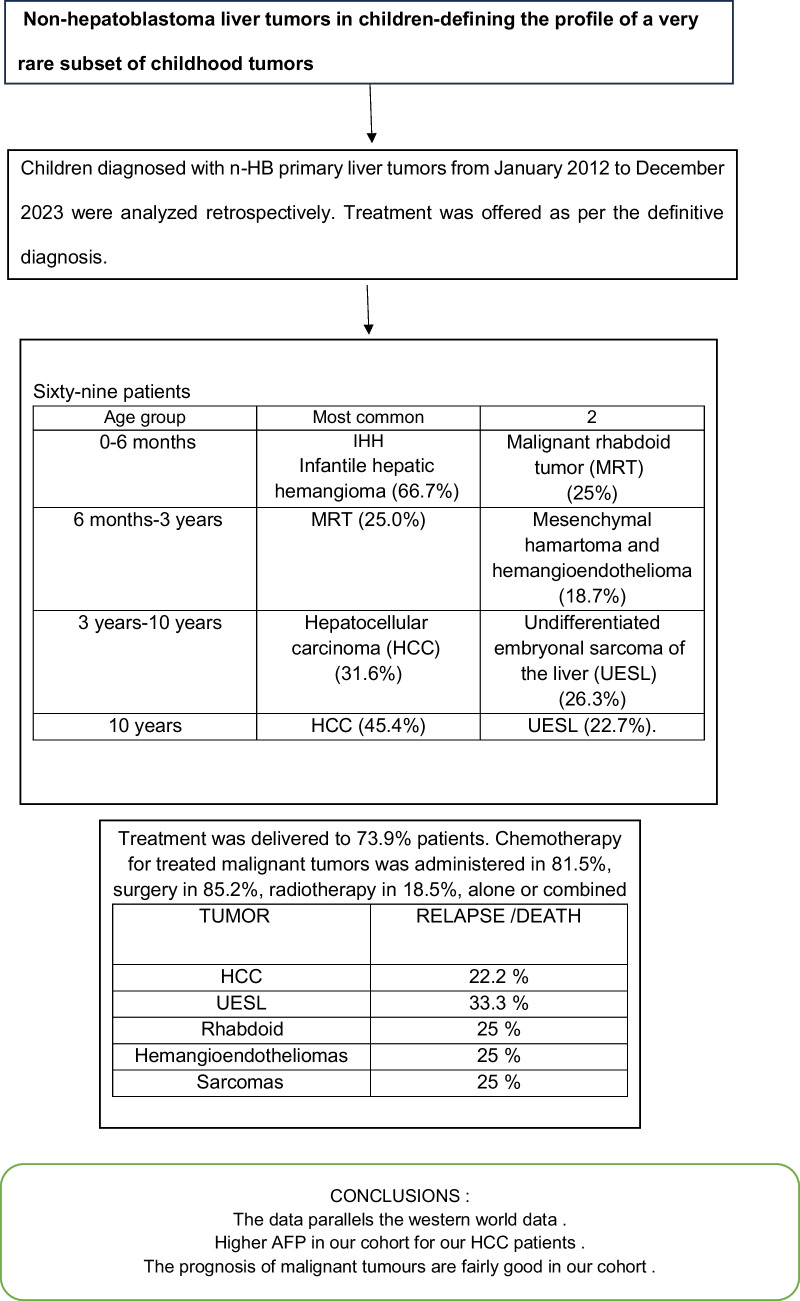

## Introduction

Liver tumors in children, although rare, present significant clinical and therapeutic challenges. Pediatric liver tumors encompass a heterogeneous group of neoplasms, with hepatoblastoma being the most common primary malignancy, followed by hepatocellular carcinoma (HCC) [1]. Hepatoblastoma and HCC accounted for 67% and 31%, respectively, of primary hepatic malignancies diagnosed in the first two decades of life according to the Surveillance, Epidemiology and End Results (SEER) database [2]. Also, hepatoblastoma represented 91% of primary liver tumors in children under five years old, while HCC constituted 87% of cases among adolescents aged 15 to 19 years [2]. Unlike adult liver cancers, which are often associated with underlying cirrhosis and chronic liver disease, pediatric liver tumors typically arise in the absence of such risk factors, suggesting distinct pathogenesis, molecular biology and clinical behavior [3]. Early detection and accurate radiological and/or histopathological classification are critical for determining prognosis and guiding treatment, which may include observation, surgical resection, chemotherapy, radiotherapy and, in selected cases, liver transplantation. The published literature available is mainly restricted to hepatoblastoma with scarce information on the epidemiology, outcomes and management of non-hepatoblastoma (n-HB) primary liver tumors. The rarity of these tumors presents a challenge for large-scale studies, emphasizing the need for collaborative research efforts and multidisciplinary care. This study aims to provide an overview of the demographic profile and outcomes for primary n-HB liver tumors in children treated over a decade at a tertiary cancer care centre.

## Methods

This is a retrospective analysis of children ≤ 15 years of age, diagnosed with primary n-HB liver tumors from January 2012 to December 2023. Diagnosis was made based on radiology, serum tumor marker levels and/or biopsy in cases wherever required. The preferred modality of local imaging was triphasic contrast-enhanced computed tomography (CECT) scan of the abdomen. Staging in case of malignant tumors was done by non-contrast computed tomography scan (NCCT) of thorax or fluorodeoxyglucose positron emission tomography computed tomography (FDG PET/CT) scan, based on the primary diagnosis. The treatment modalities included observation, chemotherapy, surgery (including liver transplantation), radiotherapy and interventional procedures including angioembolization, alone or in combination based on definitive diagnosis. Diagnostic and treatment modalities for each type of tumor followed at our institute are summarized in Table 1. The primary objective was to study the epidemiological profile of n-HB primary liver tumors in children and the secondary objectives were to delineate the treatment strategies and outcomes in them. The study was approved by the institutional ethics committee (OIEC/4709/2025/00001).

**Table 1:** Diagnostic and treatment protocol for different types of liver tumors

Type of tumor	Diagnostic modalities	Treatment modalities
IHH	Diagnosis mainly based on radiology and biopsy only if uncertain on imaging	• Observation • Propranolol (start at 1 mg/kg/day and increase to 2 mg/kg/day) • Interventional radiology procedure (angioembolisation)
HCC	Diagnosis based on pathology (biopsy or resection) Imaging of the primary and staging by triphasic CECT of abdomen, pelvis and thorax	• If resectable, upfront surgery • If borderline resectable or limited metastasis SUPERPLADO chemotherapy [4] Surgery after 4–6 cycles of NACT (hepatectomy/OLT) and metastasectomy for resectable limited metastases OLT if non-metastatic and hepatectomy not possible • If unresectable (primary or metastatic) and extensive metastases, palliation
Rhabdoid tumor	Diagnosis based on pathology (biopsy or resection), including morphology and immunohistochemistry showing loss of INI1/BRG Imaging of primary and staging by FDG-PET CECT scan	• If metastatic, palliation • If non-metastatic, chemotherapy (ICE/VDC* alternating for total 8 cycles, VDC*/VIE for the earlier cohorts) Local therapy: Surgery where R0 feasible (after 3–4 cycles of NACT) plus post-operative radiotherapy (45 Gy) Definitive radiotherapy if R0 resection not possible (55–60 Gy)
UESL	Diagnosis based on pathology (biopsy or resection) Imaging of primary and staging by CECT scan of abdomen and thorax	• Chemotherapy (4 cycles VDC, 4 cycles VIE) • Surgery after 4 cycles of NACT • ± Radiotherapy if unresectable (palliative intent)
Hemangioendothelioma	Diagnosis based on radiology and biopsy if uncertain on radiology	• Surgery • Interventional radiology procedure (angioembolisation)
Sarcoma	Diagnosis based on pathology (biopsy or resection) both morphology and immunohistochemistry (Desmin, Myogenin, Myo D1 positivity for RMS) Imaging of primary and staging by FDG-PET CECT scan in RMS, CECT scan of abdomen and thorax in other sarcomas	• Surgery • Chemotherapy depending on histology (RMS: IRS IV protocol [VAC], high grade sarcoma: IVA) • ± Radiotherapy
Germ cell tumor	Diagnosis based on elevated serum tumor marker (AFP/B HCG) levels and pathology (biopsy or resection) Imaging of primary and staging by CECT scan of abdomen and thorax	• Chemotherapy (PEb/JEb) (4 cycles for stages 1 and 2, 6 cycles for stages 3 and 4) • Surgery after 3–4 cycles of NACT

*IHH* infantile hepatic hemangioma, *HCC* hepatocellular carcinoma, *OLT* orthotopic liver transplantation, *UESL* undifferentiated embryonal sarcoma of the liver, *NACT* neoadjuvant chemotherapy, *RMS* rhabdomyosarcoma, *AFP* alpha-feto protein, *B HCG* beta human chorionic gonadotropin, *CECT* contrast-enhanced computerised tomograpy, *FDG-PET* fluoro deoxy glucose positron emission tomography

*ICE* ifosfamide iv infusion over 2 h (2000 mg/m^2^, D2 to D4) with iv mesna 400 mg/m^2^ at 0, 3, 6 and 9 h of ifosfamide, carboplatin iv infusion over 1 h (450 mg/m^2^, D1), etoposide (100 mg/m^2^, D2 to D4)

*VDC*—vincristine iv push (1.5 mg/m^2^, D1), doxorubicin iv infusion over 6 h (60 mg/m^2^, D1), cyclophosphamide iv infusion over 30 min (600 mg/m^2^, D1), *cyclophosphamide iv infusion over 30 min (1500 mg/m^2^, D1) with iv mesna 400 mg/m^2^ at 0, 3, 6 and 9 h of cyclophosphamide

*VIE*—vincristine iv push (1.5 mg/m^2^, D1), ifosfamide iv infusion over 2 h (2000 mg/m^2^ D1 to D5) with iv mesna (600 mg/m^2^ D1 to D5) at 0, 3, 6 and 9 h of ifosfamide, etoposide iv infusion over 1–2 h (100 mg/m^2^, D1 to D5)

*VAC*—vincristine iv push (1.5 mg/m^2^, D1), actinomycin-D iv push (> 3 years/1–3 years: 0.045 mg/kg, < 1 year: 0.025 mg/kg), cyclophosphamide iv infusion over 30 min (> 3 years: 2200 mg/m^2^, 1–3 years: 73 mg/kg, < 1 year: 36 mg/kg, D1) with iv mesna 500 mg/m^2^ at 0, 3, 6 and 9 h of cyclophosphamide D1

*IVA*—ifosfamide iv infusion over 2 h (2500 mg/m^2^, D1 to D3) with iv mesna (750 mg/m^2^ D1 to D3) at 0, 4 and 8 h of ifosfamide, vincristine (1.5 mg/m^2^, D1, D8), adriamycin iv infusion over 1 h (30 mg/m^2^, D1 D2)

*PEb*—cisplatin iv infusion over 2 h (20 mg/m^2^, D1 to D5), etoposide iv infusion over 1–2 h (100 mg/m^2^, D1 to D5), bleomycin iv push (15 U/m^2^, D1)

*JEb*—carboplatin iv infusion over 1 h (560 mg/m^2^, D1), etoposide iv infusion over 1–2 h (100 mg/m^2^, D1 to D5), bleomycin iv push (15 U/m^2^, D1)

## Results

### Epidemiological profile

**Fig. 1:**
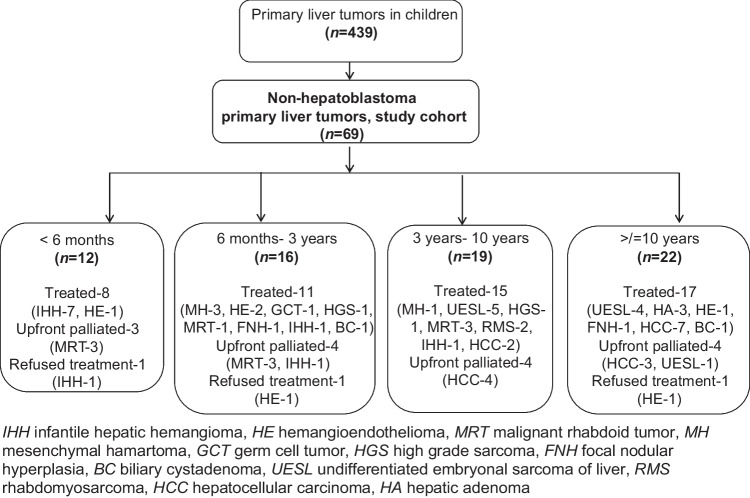
Flow diagram of the study. *IHH* infantile hepatic hemangioma, *HE* hemangioendothelioma, *MRT* malignant rhabdoid tumor, *MH* mesenchymal hamartoma, *GCT* germ cell tumor, *HGS* high-grade sarcoma, *FNH* focal nodular hyperplasia, *BC* biliary cystadenoma, *UESL* undifferentiated embryonal sarcoma of liver, *RMS* rhabdomyosarcoma, *HCC* hepatocellular carcinoma, *HA* hepatic adenoma

Primary hepatic n-HB tumors diagnosed during the study period were 69, constituting 15.7% (*n* = 69/439) of the total primary liver tumors in children. Median age was seven years (range, 0.12–15 years). Male to female ratio was 1.09:1. The ratio of malignant to benign tumors in the different age groups were < 6 months—1:3, six months to three years—1:1.6, three to 10 years—5.3:1 and >/= 10 years—2.1. Invasive procedures such as biopsy or surgery were done in 71% (*n* = 49) patients to establish a diagnosis, while radiology/tumor marker elevation clinched the diagnosis in 28.9% (*n* = 20) of the patients. The most common tumors in various age groups were zero to six months: infantile hepatic hemangioma (IHH)—66.7% (*n* = 8/12), malignant rhabdoid tumor (MRT)—25% (3/12); six months to three years: MRT—25.0% (*n* = 4/16), mesenchymal hamartoma and hemangioendothelioma—18.7% (*n* = 3/16) each; three to 10 years: hepatocellular carcinoma (HCC)—31.6% (*n* = 6/19), undifferentiated embryonal sarcoma of the liver (UESL)—26.3% (*n* = 5/19); and > = 10 years: HCC—45.4% (*n* = 10/22), UESL—22.7% (*n* = 5/22). PRE-Treatment EXTent of tumor (PRETEXT) stage in the diagnosed HCCs (*n* = 16) were I—1, II—5, II—3, IV—6 and not specified—1. A flow diagram of the study is provided in Fig. 1. Median alpha-fetoprotein (AFP) levels in ng/mL in various cancer types were HCC—131,249 (range, 2.0–1,437,700), mesenchymal hamartoma—687 (range, 5.72–2638) and hemangioma—5,823 (range, 2.0–1,374,056).

### Specific tumor profile

The clinical presentations, age group and metastatic status of the different tumor types are detailed in Table 2. Abdominal pain (63.4%, *n* = 26) and distension (43.9%, *n* = 18) were the main presenting symptoms in malignant tumors (*n* = 41). Jaundice was observed in 14.6% (*n* = 6) of the above cohort. HCC was predominantly observed in children over 10 years old, while rhabdoid tumors were more common in children under two years of age. UESL was evenly distributed between children under 10 years of age and those aged 10 years and older. Underlying cirrhosis in HCC was present in 18.7% (*n* = 3) of the patients, which was detected at the time of presentation with hepatic malignancy. Metastatic disease was present in 41.5% (*n* = 17) of the malignant tumors (*n* = 41).

**Table 2:** Clinical profile of the specific non-hepatoblastoma tumor types

Tumor type	Symptoms, *n* (%)	Age group, *n* (%)	Stage, *n* (%)	Underlying liver disease/syndrome, *n (%)*	Duration of symptoms in months, median (range)
HCC, *n* = 16	Abdominal pain—14 (87.5) Jaundice—2 (14.2) Vomiting—2 (14.2) Fever—1 (7) Weight loss—1 (7) Hematochezia—1 (7)	0–5 years—1 (7) 5–10 years—5 (35.7) > = 10 years—10 (71.4)	Metastatic—7 (43.7) (Lungs—6, lymph node—1)	Cirrhosis—3 (18.7) (Chronic hepatitis B—2, cause unknown—1)	1.25 (0.5–24)
Rhabdoid tumor, *n* = 10	Abdominal lump—9 (90) Abdominal pain—1 (10) Vomiting—1 (10) Jaundice—1 (7)	0–2 years—10 (100)	Metastatic—6 (60) (Lung—5, lymph node + brain—1)	-	0.5 (0.25–1)
UESL, *n* = 10	Abdominal pain—7 (70) Abdominal distension—5 (50) Fever—1 (10) Jaundice—1 (10)	< 10 years—5 (50) > = 10 years—5 (50)	Metastatic—2 (20) (Lung—1, lung + peritoneum—1)	-	3.5 (1–36)
Rhabdomyosarcoma, *n* = 2	Abdominal pain—2 (100) Abdominal distension—1 (50) Jaundice—1 (50)	0–5 years—2 (100)	Metastatic—1 (50) (Lymph node—1)	-	3 (1–5)
High grade sarcoma, *n* = 2	Abdominal distension—2 (100) Jaundice—1 (50) Vomiting—1 (50)	0–5 years—1 (50) 5–10 years—1 (50)	Metastatic—0	-	4.5 (2–7)
Germ cell tumor, *n* = 1	Abdominal distension—1 (100)	0–5 years—1 (100)	Metastatic—1 (100) (Lung—1)	-	0.25
Mesenchymal hamartoma, *n* = 4	Abdominal distension—4 (100) Abdominal pain—1 (25) Jaundice—1 (25)	0–2 years—2 (50) 2–5 years—1 (25) 5–10 years—1 (25)	-	-	1.75 (0.25–3)
Hemangioendothelioma, *n* = 6	Abdominal distension—5 (83.3) Hematochezia—1 (16.7) Incidental—1 (16.7) None had features of cardiac failure	0–2 years—1 (16.7) 2–5 years—3 (50) > = 10 years—2 (33.3)	Metastatic—0	-	2 (0.25–12) Excluding incidental
Hepatic adenoma, *n* = 3	Abdominal pain—2 (66.6) Abdominal distension—1 (33.3)	> = 10 years—3 (100)	-	-	0.6 (0.3–2)
Focal nodular hyperplasia, *n* = 2	Abdominal pain—1 (50) Abdominal distension—1 (50)	0–2 years—1 (50) > = 10 years—1 (50)	-	Gonadal dysgenesis—1 (50) None had portosystemic shunts	1.25 (0.25–2)
Intrahepatic biliary cystadenoma (*n* = 2)	Abdominal pain—1 (50) Abdominal distension—1 (50)	> = 10 years—2 (100)	-	-	1.5 (1–2)

*HCC* hepatocellular carcinoma, *UESL* undifferentiated embryonal sarcoma of liver

### Treatment and outcomes

Treatment was delivered to 73.9% (*n* = 51) patients. In IHH, observation was offered in 54.5% (*n* = 6/11), propranolol and surgery in 18.1% each (*n* = 2/11 each) and angioembolization in 9% (*n* = 1). Chemotherapy for treated malignant tumors (*n* = 27) was administered in 81.5% (*n* = 22), surgery in 85.2% (*n* = 23) and radiotherapy in 18.5% (*n* = 5), alone or combined.

In the different malignant cancer sub-types, the proportion of relapse/deaths in treated patients were HCC—22.2% (*n* = 2/9, relapse—2), UESL—33.3% (*n* = 3/9, relapse—2, death—1), rhabdoid—25% (*n* = 1/4, relapse—1), hemangioendotheliomas—25% (*n* = 1/4, relapse—1) and sarcomas—25% (*n* = 1/4, relapse—1). Details are in Table 3.

**Table 3:** Treatment delivered and outcomes in childhood primary non-hepatoblastoma liver tumors

Disease (Total number)	Chemotherapy	Surgery	Radiotherapy (Definitive/PORT)	Upfront palliation	Event before local control	Final outcomes in treated cohort
HCC (*n* = 16)	4 (NACT—4)	8 (hepatectomy—7, OLT—1)	0	7	1	Relapse—2 Alive at last follow-up—7
Rhabdoid tumor (*n* = 10)	4	3	1 (definitive)	6	0	Relapse—1 Alive at last follow-up—3
UESL (*n* = 10)	9	7	3 (all PORT)	1	2 (progression—1 NRM—1)	Relapse—2, death—1 Alive at last follow-up—6
Hemangioendothelioma (*n* = 6)	1 (propranolol)	1 (hepatectomy) IR embolisation—2	0	0	2 (abandoned—2)	Relapse—1 Alive at last follow-up—3
Mesenchymal hamartoma (*n* = 4)	0	4 (hepatectomy)	0	0	0	Alive at last follow-up—4
Rhabdomyosarcoma (*n* = 2)	2	2 (hepatectomy)	1 (PORT)	0	0	Alive at last follow-up—2
High grade sarcoma (*n* = 2)	2	2 (hepatectomy)	0	0	0	Relapse—1 Alive at last follow-up—1
Hepatic adenoma (*n* = 3)	0	2 (hepatectomy—1, non-anatomical resection—1) (Both were 15 cm in dimension)	0	0	0	All alive at last follow-up
Germ cell tumor (*n* = 1)	1	1 (hepatectomy)	0	0	0	Alive at last follow-up—1
Focal nodular hyperplasia (*n* = 2)	0	Non-anatomical resection—1	0	0	0	All alive at last follow-up
Intrahepatic biliary cystadenoma (*n* = 2)	0	1 (hepatectomy)	0	0	0	All alive at last follow-up

*HCC* hepatocellular carcinoma, *UESL* undifferentiated embryonal sarcoma of liver, *GCT* germ cell tumor, *PORT* post-operative radiotherapy, *NRM* non-relapse mortality

## Discussion

The existing literature on this very rare subset of primary n-NB liver tumors is scarce. A SEER cohort which included patients < 20 years of age over two-and-a-half decades reported 271 cases with primary hepatic malignancies, predominated by hepatoblastoma (67%) [2]. Primary n-HB liver tumors constituted 32.5% of the total cases, wherein HCC comprized 31% of the total cases. This publication is of an earlier cohort from 1973 to 1997 though, when rhabdoid tumor was not recognized as a specific entity and hence not reported. A recent analysis from a major liver transplant centre in India reported on 47 primary n-HB liver tumors in children over a 10-year period, with a preponderance of HCC (47%) [5]. There were no sarcomas or rhabdoid tumors reported in that cohort, probably due to a referral bias, the centre being a referral site for complex hepatic resections and liver transplantation. Our study documented nearly three-fourths of the primary liver tumors to be non-HCC, which is more than that reported in other studies. This disparity could be partly attributed to the fact that subjects were included from a recent time frame in a pediatric oncology unit, managing all types of malignancies including the newly recognized specific entities.

Most children with malignant liver tumors presented with abdominal pain and distension in our cohort, which are the common symptoms across liver malignancies, except in hepatoblastoma, where pain is typically absent unless the tumor is complicated by intra-tumoral bleeding or rupture [6, 7]. Jaundice was noted in fewer than 20% of the above cohort and data on its exact incidence in n-HB pediatric liver malignancies is limited. However, this is more observed in HCC unlike hepatoblastoma, where the liver function is usually normal [6, 7]. Serum AFP levels may be elevated in several non-hepatoblastoma liver tumors as observed in our cohort, too. Hence, a biopsy is warranted when radiologic findings or patient age are not consistent with hepatoblastoma.

Hepatocellular carcinoma predominated the primary n-HB hepatic tumors, which is concordant with previous reports. AFP was elevated in a majority of our patients with HCC (87.5%, *n* = 14/16), consistent with literature from both west and east, but slightly higher than a study from India [5]. The median AFP levels though were higher in our cohort (131,249 ng/mL) relative to what is observed in previous studies (2322–9677 ng/mL), except one which observed a higher mean AFP level of 446,927 ng/mL [6]. This elevated level in our subjects is despite a lower proportion of underlying cirrhosis (19%) in our study compared to more than 50% in the published cohorts, including a study from India with increased prevalence of tyrosinemia, which is known to be associated with elevated AFP levels [8]. This is relevant in cases when pathology cannot distinguish between hepatoblastoma and HCC because AFP levels may not provide a useful differentiation. The survival outcomes of the treated patients with HCC are comparable to the published literature, though there were more resections than transplants in our cohort, reflective of the non-cirrhotic origin of HCC [9].

A literature review of PubMed publications spanning four decades identified 34 patients with liver rhabdoid tumors, 62% of them with metastases and reported worse survival for liver site (12% survival, *n* = 4/34) [10]. This is commensurate with reported outcomes from the UK, revealing one-year survival of 14% for children with liver rhabdoid tumors [11]. Our study included 10 rhabdoid tumors of the liver, metastatic in 60% with three alive out of the treated cohort of four patients. This difference in the outcomes among the cohorts could probably be due to the selected treatment in non-metastatic subjects only in our centre. The incidence and survival of UESL in our study is similar to the published literature on a combined modality treatment protocol [12].

The study is limited in being a retrospective audit with survival data restricted by the small sample size in each sub-type of tumors. Nevertheless, this is a very rare subset treated at a single tertiary centre with data on demographic, clinical profile and outcomes. A referral bias for malignant tumors with slight underrepresentation of benign etiology is a possibility, with our centre mainly providing oncology services. This study is also limited by the unavailability of molecular and germline data in certain histological sub-types, such as rhabdoid tumors. A prospective national registry would further enhance our understanding of this ultra rare subset of liver tumors. Multicentre collaborative studies incorporating molecular and germline data could inform future therapies and strategies, especially defining the role of targeted agents in the context of pediatric liver cancers.

To conclude, the demographic profile of non-HB primary hepatic tumors in children mirrors that of the western world; however, our cohort exhibited a higher proportion of malignant tumors, particularly rhabdoid tumors and elevated median AFP levels in HCC. The overall outcomes of treated malignant tumors were relatively favorable, though limited by the sample size in this rare cohort.

## Data Availability

The data that supports these findings of the study is available from the corresponding author upon reasonable request.

## Declarations

### Disclaimer

The authors are solely responsible for the data and the contents of the paper. In no way, the Honorary Editor-in-Chief, Editorial Board Members, the Indian Society of Gastroenterology or the printer/publishers are responsible for the results/findings and content of this article.
